# Vitamin D receptor gene polymorphism and polycystic ovary syndrome susceptibility

**DOI:** 10.1186/s12920-023-01541-8

**Published:** 2023-05-18

**Authors:** Ibrahim A Albahlol, Mustafa Neamatallah, Mohamed Saad Serria, Abdel-Hady El-Gilany, Yomna A Setate, Nashwa M. Alkasaby, Sally Abdallah Mostafa, Mahmoud Abdelaziz, Hossam Elazab, Omar A. Ammar

**Affiliations:** 1 Department of Obstetrics and Gynecology, Jouf College of Medicine, Jouf University, Mansoura College of Medicine, Mansoura University, Mansoura, SA Egypt; 2 Department of Medical Biochemistry and Molecular Biology, Faculty of Medicine, Mansoura University, Mansoura, Egypt; 3 Department of Public Health and Preventive Medicine, Faculty of Medicine, Mansoura University, Mansoura, Egypt; 4 Infection Control Unit, Mansoura Specialized Hospital (MSH), Mansoura, Egypt; 5 Departments of Medical Microbiology & Immunology, Faculty of Medicine, Mansoura University, Mansoura, Egypt; 6 Oncology Center, Mansoura University, Mansoura, Egypt; 7 Basic Science Department, Delta University for Science and Technology, Gamasa, Egypt

**Keywords:** PCOS, Vitamin D receptor, Gene polymorphisms, SNP

## Abstract

**Background:**

Polycystic ovary syndrome (PCOS) is the most common endocrinopathy in women. This study was designed to investigate the associations of vitamin D receptor (VDR) gene variants with PCOS risk and the severity of the disease phenotype among Egyptian women.

**Methods:**

In this study, 185 women with PCOS and 207 fertile women as controls were recruited. Cases were divided into phenotype groups based on their clinical and paraclinical features. Clinical and laboratory data were measured in the patient and control groups. All individuals were genotyped for nine single-nucleotide polymorphisms (SNPs) located across the VDR gene using Taq^Man^ allelic discrimination real-time polymerase chain reaction.

**Results:**

Women with PCOS were significantly (*P* ≤ 0.001) higher body mass index (BMI) (22.77 ± 2.5) than controls (21.68 ± 1.85 kg/m^2^). Women with PCOS had significantly higher anti-Mullerian hormone, prolactin, luteinizing hormone (LH), LH/follicle-stimulating hormone (FSH), free testosterone, total testosterone, and dehydroepiandrosterone sulfate levels than the control group (*P* ≤ 0.001). The level of FSH was significantly lower in women with PCOS than in the control group (*P* ≤ 0.001). Analysis of the VDR rs4516035, rs2107301, rs1544410 (BsmI), and rs731236 (TaqI) SNPs showed a significant association with PCOS phenotype A. Furthermore, rs2228570 (FokI), rs3782905, rs7975232 (ApaI), and rs739837 SNPs showed a significant association with PCOS phenotype C. Furthermore, rs11568820 SNP showed a significant association with PCOS phenotype D (*P* < 0.05).

**Conclusions:**

The findings of this study indicate that variations in the VDR gene were associated with an increased risk of PCOS in Egyptian women.

## Introduction

Polycystic ovary syndrome (PCOS) is a prevalent endocrine disorder that affects 6-12% of women of reproductive age worldwide [1]. The syndrome is a complicated and diverse disorder with a variety of clinical symptoms, the most common of which are hyperandrogenism and/or hyperandrogenemia (HA), oligo/anovulation (OA), and polycystic ovary morphology (PCOM) [2, 3]. PCOS women are divided into four phenotypes: phenotype A (HA, OA, and PCOM), phenotype B (HA and OA), phenotype C (HA and PCOM), and phenotype D (OA and PCOM) [4].

A significant potential gene for PCOS is the vitamin D receptor (VDR) gene, also known as the calcitriol receptor or NR1I1 [5]. It is a ligand-activated transcription factor that mediates the genomic effects of vitamin D and controls a number of endocrine and cellular processes, such as bone metabolism and calcium-phosphate homeostasis [6]. Several tissues, including the skeletal, parathyroid, and reproductive systems, express it, and several target genes are modulated to induce a range of biological consequences. The VDR gene has a chromosomal location of 12q12–14 containing a promoter region that is pre-extensive and able to produce many transcripts that are tissue-specific. Research has been done on several VDR polymorphisms to determine their functional importance and possible impacts on disease susceptibility to complicated diseases such as osteoarthritis (OA), diabetes, cancer, high myopia, cardiovascular disease, and tuberculosis [7–12]. A few studies have also looked at the role of the VDR gene in endocrine diseases such as PCOS [6, 13, 14].

Approximately, 200 single-nucleotide polymorphisms (SNPs) of the VDR gene were found to be involved [15]. Vitamin D and VDR variations, including Cdx2, Fok1, Apa1, and Taq1, have been linked to the endocrine, metabolic, and genetic components of PCOS, demonstrating their critical functional involvement [6, 14, 16].

This study was designed to examine the association of the nine functionally most relevant VDR SNPs rs11568820 (C/T), rs4516035 (C/T), rs3782905 (C/G), rs2228570 (FokI) (C/T), rs2107301 (C/T), rs1544410 (BsmI) (G/A), rs7975232 (ApaI) (A/C), rs731236 (TaqI) (T/C), and rs739837 (T/G) with PCOS risk and the severity of the disease phenotype among Egyptian women.

## Patient and method

### Study population

This case-control study included a convenience sample of 392 women (207 healthy controls and 185 cases of PCOS) recruited at the Obstetrics and Gynecology Department, Mansoura University Hospital, Egypt. Laboratory workup was performed at the Molecular Genetic Unit in Endemic Hepatogastroenterology and Infectious Diseases, Faculty of Medicine, Mansoura University, during the period from January 2014 to November 2016.

The subjects were classified according to the Rotterdam consensus (Rotterdam ESHRE/ASRM Sponsored PCOS consensus workshop group, 2004). PCOS was diagnosed according to the Rotterdam criteria [4]. The first group consisting of 207 healthy fertile women without any PCOS features and who have regular menstrual cycles (26–35 days) was enrolled as the control group. The second group comprised 185 women with PCOS, including 88 with phenotype A PCOS (OD + HA + PCOM), 41 with phenotype C PCOS (HA + PCOM), and 56 with phenotype D PCOS (OH + PCOM).

All participants were females of reproductive age who had not been pregnant and had not received hormone treatment for at least three months prior to the study. Age and body mass index (BMI), which is determined by dividing weight (kg) by height^2^ (m^2^), were reported to exclude obese women. Follicle-stimulating hormone (FSH), luteinizing hormone (LH), total testosterone (TT), and free testosterone (FT) were detected by chemiluminescence immunization (Beckman Access Health Company, Chaska, MN, USA) with intra- and inter-assay variation coefficients of 10% during the early follicular phase of the cycle. Several recognized causes of androgen excess and ovulatory dysfunction were excluded. Serum cholesterol and triglycerides (TG) were evaluated in the fasting blood samples using precipitation and enzymatic methods.

### Genomic DNA extraction from peripheral blood

All individuals had their genomic DNA extracted from peripheral blood using a commercial Qiagen DNA isolation kit (QIAmp DNA Mini kit; Qiagen, Hilden’s, Germany), following the manufacturer’s instructions. A 2% ethidium bromide-stained agarose gel was used to determine the integrity of the DNA using a NanoDrop spectrophotometer (NanoDrop^TM^2000/2000c, Thermo-Fisher Scientific, CA, USA).

### Detection of VDR polymorphisms

**Fig. 1:**
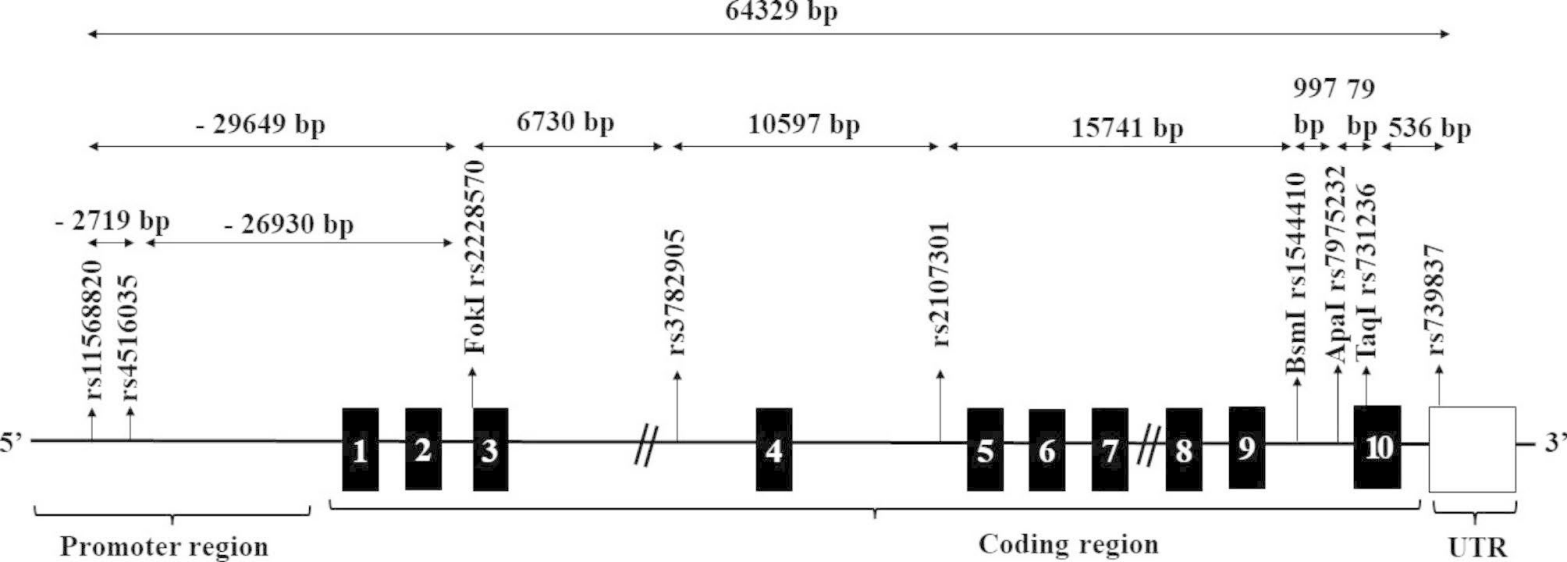
Arrangement of proposed restriction sites within the VDR’s genetic region. The exons of the VDR gene are shown by the black boxes. Arrows are used to indicate where possible polymorphisms are located

Nine SNPs located in the entire VDR gene were selected for the current study (Fig. 1). These SNPs include two SNPs namely, 1- rs11568820 (C/T) and 2- rs4516035 (C/T) in the promoter region flanking to transcriptional region. Six SNPs located in the transcriptional region; 3- rs2228570 (FokI) (C/T), 4- rs3782905 (C/G), 5- rs2107301 (C/T), 6- rs1544410 (BsmI) (G/A), 7- rs7975232 (ApaI) (A/C), 8- rs731236 (TaqI) (T/C), and 9- rs739837 (T/G) located in untranslated region (UTR). TaqMan allelic discrimination SNP primers were made. Fluorescent dyes were used to mark the probes that were particular to each allele (VIC and FAM). Probes are used in a real-time PCR reaction on a device (Applied Biosystems, model 7500) to type each DNA sample’s allele. Fluorescein-amidite-labeled SNP primers and probes that are already produced for use in real-time PCR (purchased from Applied Biosystems).

The following 20 µL were used for the reaction: 10.0 µL Universal Master Mix II by TaqMan (2×), 1.0 µL Assay Mix for SNP Genotyping (20×), 1.0 µL template for DNA, and 8.0 µL of RNase free water. A thermal cycler was used to carry out real-time PCR genotyping (Applied Biosystems, 7500 Real-Time PCR system). The subsequent cycles were utilized: the first step in denaturation at 95ºC for 10 min, then the denaturation stage for 40 cycles at 95ºC for 15 s, and the stage of annealing/extension at 60ºC for 1 min. Taq^Man^ plate well fluorescence intensities were measured. Applied Biosystems, Foster City, California, USA’s automated allele-calling software (SDS 2.4) was used to evaluate the fluorescence data files from each plate [17]. To confirm the quality of the study, duplicate genotypes were performed on 10% of all samples. For statistical analysis, SPSS software was used to export all genotyping data.

### Statistical analyses

Statistical analysis was performed on the data using the SPSS software program (IBM Corp. Released 2012, Version 21.0. IBM SPSS Statistics for Windows; Armonk, NY, USA). Numbers and percentages were used to convey categorical data The categorical data were compared using the chi-square or Monte Carlo test if required. The Shapiro test was used to determine whether quantitative data were normal. The mean and SD were utilized as variables with a normal distribution. More than two groups were compared using one-way ANOVA with Bonferroni post hoc multiple comparisons. The non-parametric variables were described using the median and interquartile ranges. In contrast to the Mann-Whitney test, which was used to compare just two groups, the Kruskal-Wallis test was used to compare several groups. Each allele’s total genotyping was scored and totaled in each group. Allele carriage is the proportion of people who carry at least one variation of a certain allele. A given allele’s odds ratio (OR) and 95% confidence interval (CI) were computed, compared to the case in which the target allele is not carried, using the program Med Calc (Med Calc statistical software version 16.4.3). Each SNP’s allele carriage variables were compared using chi-square and Fisher’s exact tests. The difference was thought to be significant if *P* < 0.05.

## Results

The clinical characteristics of the PCOS and fertile groups and the PCOS phenotype groups, including PCOS phenotype, age, pre-pregnancy BMI, gravity, parity, and primary and secondary infertility data, are shown in Table 1. Women with PCOS were significantly older (24.09 ± 3.84 years vs. 21.57 ± 3.63 years) and had higher BMI (22.77 ± 2.5 kg/m^2^ vs. 21.68 ± 1.85 kg/m^2^) than the control group.

**Table 1:** Clinical characteristics of the fertile and PCOS groups

	Fertile (207) Mean ± SD	PCOS (185) Mean ± SD	PCOS phenotypes		
			A (88) Mean ± SD	C (41) Mean ± SD	D (56) Mean ± SD
**Age (Years)**	21.57 ± 3.63	24.09 ± 3.84^***^	23.22 ± 3.67^***^	24.93 ± 3.64^***^	24.84 ± 4.0^***^
**Pre-pregnancy BMI (kg/m** ^**2**^ **)**	21.68 ± 1.85	22.77 ± 2.5^***^	22.6 ± 2.23^***^	22.8 ± 2.8^***^	23.0 ± 2.71^***^
**Gravity, n(%)**					
**1**	103(49.76)	98(52.97)	45(51.14)	24(58.54)	29(51.79)
**2**	65(31.4)	62(33.51)	27(30.68)	14(34.15)	21(37.5)
**3**	30(14.49)	19(10.27)	15(17.05)	1(2.44)	3(5.36)
**4**	8(3.86)	6(3.24)	1(1.14)	2(4.88)	3(5.36)
**5**	1(0.48)	0	0	0	0
**Parity, n(%)**					
**0**	15(7.25)	128(69.19)	63(71.59)	28(68.29)	37(66.07)
**1**	114(55.07)	45(24.32)	17(19.32)	12(29.27)	16(28.57)
**2**	57(27.54)	12(6.49)	8(9.09)	1(2.44)	3(5.36)
**3**	18(8.7)	0	0	0	0
**4**	3(1.45)	0	0	0	0
**Infertility, n(%)**					
**Primary**	0	98(52.97)	45(51.14)	24(58.54)	29(51.79)
**Secondary**	0	87(47.03)	43(48.86)	17(41.46)	27(48.21)

PCO, polycystic ovary; SD, standard deviation; BMI, body mass index, ^**, ***^ significant difference compared with the fertile group at P ≤ 0.01 and P ≤ 0.001, respectively, using the t-test.

Laboratory data of the fertile and PCOS groups and the PCOS phenotype groups, including anti-Mullerian hormone (AMH), prolactin, LH, FSH, LH/FSH, FT, TT, dehydroepiandrosterone sulfate (DHEA-s), cholesterol, TG, and HbA1c levels, are shown in Table 2. Women with PCOS had significantly higher AMH, prolactin, LH, LH/FSH, FT, TT, and DHEA-s levels than the fertile group (*P* ≤ 0.001). Furthermore, women with PCOS had significantly higher levels of cholesterol, TG, and HbA1c than the controls (*P* ≤ 0.001). The level of FSH was significantly lower in women with PCOS than in the controls (*P* ≤ 0.001).

**Table 2:** Laboratory data of the fertile and PCOS groups and the PCOS phenotype groups

	Fertile (207) Mean ± SD	PCOS (185) Mean ± SD	PCOS phenotypes		
			A (88) Mean ± SD	C (41) Mean ± SD	D (56) Mean ± SD
**AMH (ng/ml)**	5.1 ± 0.78	9.77 ± 1.8^***^	9.44 ± 1.29^***^	8.58 ± 1.19^***^	11.16 ± 1.98^***^
**Prolactin (ng/dl)**	9.75 ± 2.75	15.54 ± 5.49^***^	16.06 ± 5.88^***^	15.93 ± 5.1^***^	14.43 ± 5.05^***^
**LH (IU/L)**	6.73 ± 2.48	11.16 ± 4.45^***^	13.02 ± 4.38^***^	10.88 ± 4.34^***^	8.45 ± 3.03^***^
**FSH (IU/L)**	7.32 ± 2.68	4.15 ± 1.44^***^	4.55 ± 1.43^***^	4.35 ± 1.53^***^	3.39 ± 1.07^***^
**LH/FSH**	0.92 ± 0.16	2.67 ± 0.53^***^	2.86 ± 0.43^***^	2.49 ± 0.51^***^	2.51 ± 0.59^***^
**Free testosterone (ng/dl)**	0.21 ± 0.12	0.85 ± 0.27^***^	0.89 ± 0.29^***^	0.9 ± 0.24^***^	0.75 ± 0.23^***^
**Total testosterone (ng/dl)**	46.04 ± 26.45	184.19 ± 58.22^***^	192.82 ± 62.17^***^	194.72 ± 52.67^***^	162.92 ± 50.47^***^
**DHEA-s (µg/ml)**	1.75 ± 0.47	3.1 ± 0.75^***^	3.32 ± 0.77^***^	3.33 ± 0.717^***^	2.59 ± 0.41^***^
**Cholesterol (mg/dl)**	159.26 ± 15.17	190.95 ± 14.74^***^	196.85 ± 13.77 ^***^	185.51 ± 14.65^***^	185.64 ± 12.83^***^
**TG (mg/dl)**	105.15 ± 22.43	115.68 ± 14.74^***^	117.58 ± 28.15^***^	113.27 ± 19.12^***^	114.46 ± 14.64^***^
**HbA1c**	5.44 ± 0.5	5.66 ± 0.53^***^	5.64 ± 0.52^**^	5.71 ± 0.56^**^	5.66 ± 0.52^**^

PCO, polycystic ovary; SD, standard deviation; AMH, anti-Mullerian hormone; LH, luteinizing hormone; FSH, follicle-stimulating hormone; DHEA-s, dehydroepiandrosterone sulfate; TG, triglycerides; HbA1c, hemoglobin A1C. ^**, ***^ significant difference compared with the fertile group at P ≤ 0.01 & P ≤ 0.001, respectively, using the t-test.

The different genotypes of PCOS phenotypes and the fertile group of the VDR gene SNPs are shown in Table 3. In this study, two SNPs located in the promoter region, namely, rs11568820 and rs4516035, were genotyped. The minor allele frequencies were 0.15 and 0.16 in the fertile group compared with those (0.47 and 0.28) in the PCOS group, respectively. Furthermore, six SNPs located in the transcriptional region, namely, rs2228570 (FokI), rs3782905, rs2107301, rs1544410 (BsmI), rs7975232 (ApaI), and rs731236 (TaqI), were genotyped. The minor allele frequency of rs2228570 (FokI) initial codon was 0.16 in the fertile group, whereas that in the PCOS groups was 0.28. Meanwhile, the minor allele frequencies of rs3782905 and rs2107301 in introns 3 and 4 were 0.14 and 0.11 in the fertile group, whereas those in the PCOS group were 0.29 and 0.26, respectively.

Furthermore, the minor allele frequencies of rs1544410 (BsmI) and rs7975232 (ApaI) located in intron 9 were 0.15 and 0.21 in the fertile group, whereas those in the PCOS group were 0.30 and 0.32, respectively. Meanwhile, the minor allele frequency of rs731236 (TaqI) located in exon 10 was 0.12 in the fertile group, whereas that in the PCOS group was 0.32. Finally, rs739837 SNP located in the UTR region was genotyped. The minor allele frequency was 0.13 in the fertile group, whereas that in the PCOS group was 0.28. The differences in minor allele carriage of different SNPs were analyzed in the PCOS and PCOS phenotype groups and compared with those in the fertile group using the autosomal dominant model. The total minor allele frequency ranged from 0.18 to 0.30, and the global minor allele frequency ranged from 0.18 to 0.49. The distribution of allele carriages was tested for equilibrium using the Hardy–Weinberg equation. The allele carriages of all SNPs in all groups were balanced (*P* > 0.05). The allele carriages and frequencies of the sub-phenotypes of PCOS are also shown in Table 3. No significant difference was observed between the allele carriage of primary infertility and that of secondary infertility.

**Table 3:** The genotype distribution and allele frequency of VDR SNPs in the fertile and PCOS groups and the PCOS phenotype groups, 1^ry^ infertility and 2nd infertility

SNP (AJ/AI)	Genotypes M/H/N								MAF	HW		GMAF
	Fertile (207)	PCOS (185)	PCOS phenotypes			Total (392)	1^ry^ infertility	2nd infertility				
			A (88)	C (41)	D (56)					*q* ^*2*^	*P*	
**rs11568820** (**C/T)**	154/45/8 (0.15)	63/71/51 (0.47)	31/30/27 (0.48)	14/16/11 (0.46)	18/25/13 (0.45)	217/116/59	31/41/26	32/30/25	0.30	3.76	0.052	0.46
**rs4516035 (C/T)**	149/49/9 (0.16)	120/27/38 (0.28)	54/16/18 (0.30)	26/7/8 (0.28)	40/4/12 (0.25)	269/76/47	60/20/18	60/7/20	0.22	3.36	0.066	0.18
**rs2228570 (FokI) (C/T)**	146/54/7 (0.16)	110/48/27 (0.28)	56/22/10 (0.24)	21/12/8 (0.30)	33/14/9 (0.29)	256/102/34	56/29/13	51/22/14	0.22	0.52	0.473	0.33
**rs3782905 (C/G)**	154/48/5 (0.14)	104/55/26 (0.29)	53/26/9 (0.25)	22/11/8 (0.33)	29/18/9 (0.32)	258/103/31	53/33/12	51/22/14	0.21	0.29	0.532	0.24
**rs2107301 (C/T)**	165/37/5 (0.11)	108/58/19 (0.26)	48/29/11 (0.29)	24/15/2 (0.23)	36/14/6 (0.23)	273/95/24	60/29/9	48/29/10	0.18	2.59	0.107	0.34
**rs1544410 (BsmI) (G/A)**	153/47/7 (0.15)	104/51/30 (0.30)	46/28/14 (0.32)	25/9/7 (0.28)	33/14/9 (0.29)	257/98/37	51/28/19	53/23/11	0.22	1.92	0.165	0.30
**rs7975232 (ApaI) (A/C)**	133/60/14 (0.21)	99/54/32 (0.32)	52/23/13 (0.28)	18/14/9 (0.39)	29/17/10 (0.33)	232/114/46	49/30/19	50/24/13	0.26	3.72	0.052	0.45
**rs731236 (TaqI) (T/C)**	164/38/5 (0.12)	102/48/35 (0.32)	44/28/16 (0.34)	23/9/9 (0.33)	35/11/10 (0.28)	266/86/40	53/25/20	49/23/15	0.21	2.26	0.133	0.28
**rs739837 (T/G)**	159/42/6 (0.13)	107/51/27 (0.28)	50/24/14 (0.30)	21/12/8 (0.34)	36/15/5 (0.22)	266/93/33	59/25/14	52/22/13	0.20	2.31	0.129	0.49

SNP, single-nucleotide polymorphism; AJ, major allele; AI, minor allele; M/H/N, number of homozygous individual carriage major alleles/number of heterozygotes/number of minor allele homozygotes; PCOS, polycystic ovary syndrome; MAF, minor allele frequency; HW, Hardy–Weinberg equilibrium; *P*, probability < 0.05; GMAF, global minor allele frequency.

**Fig. 2:**
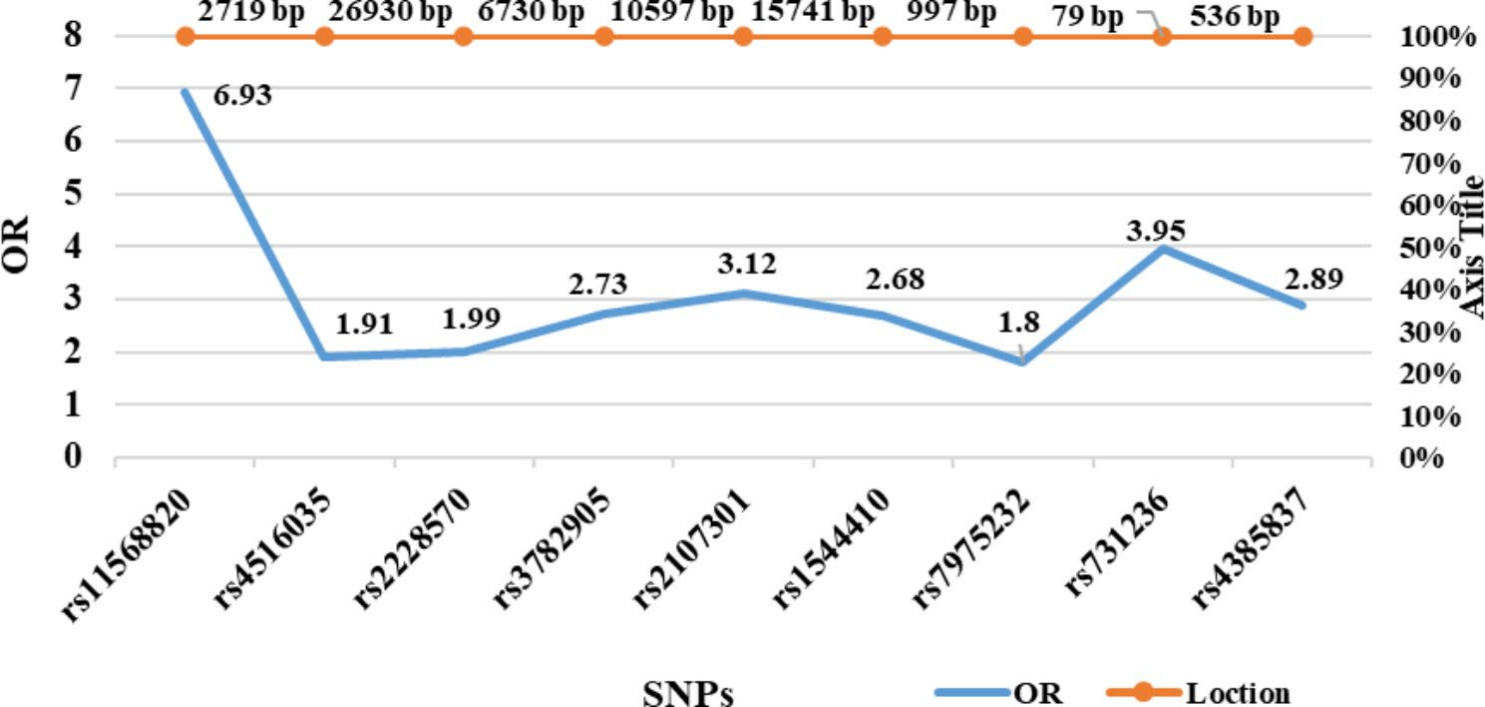
Statistical analyses of the odds ratio of the association of VDR SNPs with PCOS and the location of the candidate SNPS.

The risk of minor allele carriage of different SNPs was compared in different phenotypes and compared with that in the fertile group using a dominant model, which means that at least one copy of a minor allele is associated with the disease (Table 4). The carriage of the T allele of rs11568820 SNP located in the promoter region had the highest association in the PCOS group compared with that of the control group (OR, 6.93; 95% CI, 4.58–10.48; *P* < 0.0001) (Fig. 2). The same results were obtained for the PCOS phenotype groups as a highly significant risk association was observed between phenotypes A, C, and D compared with that in the control group (*P* < 0.0001). The association of the T allele carriage of rs4516035 located in the promoter region was also higher among the PCOS group and phenotypes A and C than that in the control group.

Along the transcriptional region, a significant association of the T allele carriage of rs2228570 (FokI) located in the initial codon was observed among the PCOS group and phenotypes C and D compared with that in the control group. Furthermore, there was a significant association between the G allele carriage of rs3782905, which is located in intron 3, and the T allele carriage of rs2107301, which is located in intron 4, among the PCOS group and all PCOS phenotype groups. In intron 9, there was a significant association of the A allele carriage of rs1544410 (BsmI) among the PCOS group and all PCOS phenotype groups and the C allele carriage of rs7975232 (ApaI) among the PCOS group and phenotypes C and D only. Furthermore, there was a significant association between the C allele carriage of rs731236 (TaqI) located in exon 10 among the PCOS group and all PCOS phenotype groups. Finally, there was a significant association between the G allele carriage of rs739837 located in the UTR region among the PCOS group and all PCOS phenotype groups.

**Table 4:** Comparisons of VDR SNPs between the fertile and PCOS groups

SNP		Fertile vs. PCOS	Fertile vs. phenotype A	Fertile vs. phenotype C	Fertile vs. phenotype D
**rs11568820**	OR 95% CI *P*	6.93 4.58 to 10.48 < 0.0001	6.84 4.11 to 11.36 < 0.0001	6.85 3.47 to 13.54 < 0.0001	7.15 3.87 to 13.21 < 0.0001
**rs4516035**	OR 95% CI *P*	1.91 1.29 to 2.82 0.0012	2.14 1.33 to 3.45 0.0018	1.97 1.05 to 3.7 0.0355	1.56 0.89 to 2.73 0.1230
**rs2228570 (FokI)**	OR 95% CI *P*	1.99 1.34 to 2.95 0.0006	1.61 0.98 to 2.64 0.0581	2.86 1.52 to 5.4 0.0012	2.08 1.18 to 3.66 0.0110
**rs3782905**	OR 95% CI *P*	2.73 1.82 to 4.1 < 0.0001	2.2 1.34 to 3.64 0.0020	3.26 1.72 to 6.17 0.0003	2.55 1.38 to 4.69 0.0027
**rs2107301**	OR 95% CI *P*	3.12 2.04 to 4.77 < 0.0001	3.73 2.24 to 6.21 < 0.0001	2.78 1.4 to 5.51 0.0034	2.54 1.39 to 4.62 0.0024
**rs1544410 (BsmI)**	OR 95% CI *P*	2.68 1.8 to 3.99 < 0.0001	3.05 1.87 to 4.98 < 0.0001	2.31 1.22 to 4.37 0.0104	2.43 1.38 to 4.3 0.0022
**rs7975232 (ApaI)**	OR 95% CI *P*	1.8 1.23 to 2.63 0.0024	1.42 0.89 to 2.29 0.1438	2.69 1.42 to 5.08 0.0024	1.93 1.11 to 3.36 0.0206
**rs731236 (TaqI)**	OR 95% CI *P*	3.95 2.61 to 6.0 < 0.0001	4.66 2.81 to 7.72 < 0.0001	4.01 2.11 to 7.63 < 0.0001	3.03 1.69 to 5.41 0.0002
**rs739837**	OR 95% CI *P*	2.89 1.92 to 4.35 < 0.0001	3.06 1.86 to 5.03 < 0.0001	3.93 2.06 to 7.48 < 0.0001	2.04 1.13 to 3.71 0.0187

SNP, single-nucleotide polymorphism; PCOS, polycystic ovary syndrome; OR, odds ratio; CI, confidence interval; *P*, probability < 0.05. The highlighted cells indicate the highest significant risk.

## Discussion

A popular genetic approach for examining relationships between candidate genes and dichotomous disease features is the unrelated case-control design. The probed SNP may be in high linkage disequilibrium with a causal variant or the SNP itself may be a common variant that significantly affects the trait if the association is significant. Population stratification within unrelated case-control populations, however, is a potential issue that should be taken into account in studies that integrate data. A family-based case-control design is an alternate strategy to prevent spurious association [18, 19]. However, the availability of a nuclear family with an index case and one fertile case is very difficult. Alternatively, genotyping adjacent markers may help eliminate spurious association in an unrelated case-control study [20]. In this study, nine markers spanning ~ 65.0 kb on the VDR gene, including promoter, exons, introns, and UTR regions, were detected.

Patients with PCOS present with alterations in sex hormone production and cholesterol and TG levels. The results showed a significant association of PCOS with BMI, AMH, prolactin, LH, FSH, FT, TT, and DHEA-s levels. When the hormone levels were compared, the level of LH was significantly higher in the PCOS group than in the control group, whereas FSH levels were significantly lower. However, the LH/FSH ratio was significantly higher in the PCOS group than in the control group. These results agree with those of many studies [21, 22].

PCOS is a multigenic disease with several causes, where several genes interact with one another as well as with environmental conditions, impacting the syndrome’s development and symptoms. Numerous biological systems rely on vitamin D and It uses VDR to moderate its activities. VDR is found in a variety of tissues, including pancreatic beta cells, skin, parathyroid, pituitary gland, or reproductive tissue, participating in the regulation of several endocrines, metabolic or reproductive functions [23]. SNPs in the VDR gene have been linked to key metabolic and endocrine parameters in PCOS, according to genetic association studies [24]. The selected SNPs were (rs11568820, rs4516035, rs2228570 (FokI), rs3782905, rs2107301, rs1544410 (BsmI), rs7975232 (ApaI), rs731236 (TaqI), and rs739837) were examined among women with PCOS and in healthy control, fertile women, the carriage of minor allele frequency of each SNP was tested for their association with the PCOS and its phenotypes compared to that of fertility group. Our interest in this research of PCOS patients arose from the fact that several studies have demonstrated the vital roles that VDR plays in the female reproductive system.

The transcription factor rs11568820 controls the transcription of the VDR gene, mRNA, and protein level [25, 26]. Yamamoto et al. [27] were the first to find an effective binding site in the 1a promoter region of the VDR gene for the intestinal-specific transcription factor rs11568820. the change from C to T, which was investigated in our study, was initially explained by Arai et al. [25] and was discovered to alter the VDR gene’s transcription in the intestine. The A-allele is more precisely bound by the CDX2 protein, which increases the transcription of the VDR gene. It should be noted that the VDR is a transcription factor and controls the transcription of genes important for glucose metabolism and other downstream genes in numerous tissues [26, 28]. Dasgupta et al. concluded that instances of the rs11568820 GA genotype and A allele were substantially more common compared to controls and seemed to provide defense against developing PCOS [29].

Statistics from a prior meta-analysis showed that VDR ApaI (rs7975232) and VDR BsmI (rs1544410) polymorphisms are linked to PCOS susceptibility in the Asian population [30]. Mahmoudi and coworkers [31] found that the ApaI (rs7975232) genotype AA might be used as a marker of reduced vulnerability to PCOS, while the CC genotype was linked to a higher incidence of PCOS. In contrast, Bagheri et al. [32] found no statistically significant correlation between that finding and PCOS risk. Iranian research found a link between the VDR TaqI (rs731236) (CC) genotype and blood LH levels [6]. This discovery was supported by Bagheri and coworkers [5]. Based on ethnic variety, only a few elevated PCOS risks were seen among Asians with the BsmI G/A polymorphism. In addition, TaqI T/C and FokI C/T polymorphisms for PCOS risk did not show any significant association, except for a few sporadic instances of elevated PCOS risk in the first in recessive models. A previous meta-analysis suggested that VDR gene polymorphisms have a role in the development of PCOS, particularly in Asian populations [33]. In the Asian population but not in the Caucasian population, VDR ApaI (rs7975232) and VDR BsmI (rs1544410) SNPs were substantially related with PCOS susceptibility, according to the analysis of subgroups by ethnicity. Genetic differences between the various ethnic groups might be the cause of this finding. Different groups may have some variances in the functional variants as a result of the process of natural selection [30].

In conclusion, this study suggests that VDR gene polymorphisms can be a good candidate for PCOS development among the Egyptian population. Further case-control studies on various ethnic populations with a larger sample size are needed to verify the current conclusions in the future.

## Data Availability

The datasets used and analyzed during this study are available from the corresponding author upon reasonable request.
